# Quorum Sensing Activity of *Enterobacter asburiae* Isolated from Lettuce Leaves

**DOI:** 10.3390/s131014189

**Published:** 2013-10-22

**Authors:** Yin Yin Lau, Joanita Sulaiman, Jian Woon Chen, Wai-Fong Yin, Kok-Gan Chan

**Affiliations:** Division of Genetics and Molecular Biology, Institute of Biological Sciences, Faculty of Science, University of Malaya, Kuala Lumpur 50603, Malaysia; E-Mails: yinyinlau88@yahoo.com (Y.Y.L.); joanitasulaiman@yahoo.com (J.S.); cjw246@hotmail.com (J.W.C.); yinwaifong@yahoo.com (W.-F.Y.)

**Keywords:** *Enterobacter asburiae*, MALDI-TOF, mass spectrometry, *N*-acyl homoserine lactone, *N*—hexanoyl homoserine lactone, *N*-butanoyl homoserine lactone, quorum sensing, lettuce, food spoilage, food microbiology, food safety

## Abstract

Bacterial communication or quorum sensing (QS) is achieved via sensing of QS signaling molecules consisting of oligopeptides in Gram-positive bacteria and *N*-acyl homoserine lactones (AHL) in most Gram-negative bacteria. In this study, *Enterobacteriaceae* isolates from Batavia lettuce were screened for AHL production. *Enterobacter asburiae*, identified by matrix-assisted laser desorption ionization time of flight mass spectrometry (MALDI-TOF-MS) was found to produce short chain AHLs. High resolution triple quadrupole liquid chromatography mass spectrometry (LC/MS) analysis of the *E. asburiae* spent supernatant confirmed the production of *N*-butanoyl homoserine lactone (C4-HSL) and *N*–hexanoyl homoserine lactone (C6-HSL). To the best of our knowledge, this is the first report of AHL production by *E. asburiae*.

## 1. Introduction

Quorum sensing (QS) refers to the capabilities of microorganisms to communicate via secreted signaling molecules called autoinducers which contribute to the regulation of gene expression in response to surrounding bacterial population density [1]. In general, Gram-negative bacteria use AHLs as autoinducers [2]. AHLs consist of 4- to 18-carbon side chains linked to a lactone ring [3]. The molecules are synthesized by the activity of LuxI synthase using *S*-adenosylmethionine (SAM) and acylated acyl carrier protein (Acyl-ACP) as substrates [4]. Gram-negative bacteria employ AHL as QS signals in their communication circuits to regulate a diverse array of physiological activities. These processes include symbiosis, virulence, competence, conjugation, antibiotic production, motility, sporulation, and biofilm formation [2].

The nutrient contents of vegetables and fruits are suitable for the growth of Gram-negative bacteria, particularly *Enterobacteriaceae* [5,6], resulting in accelerated spoilage as well as cases of food poisoning [7,8]. Studies on the microbial flora in vegetables and fruits indicate competitive inhibition within the microbial community which may affect food quality [9]. It is believed that this interaction among the microorganisms, and in particular, the availability of AHL-regulated systems may be responsible for the deterioration, toxicity and the ultimate safety of food products [10].

*Enterobacter asburiae* is a Gram-negative bacillus belonging to the *Enterobacteriaceae* family that has been isolated from soil, water and food products [11,12]. It is termed an epiphytic bacterium [13], which are generally described as microorganisms that live on plant surfaces and can either be favorable or harmful to the plant they reside on [14]. Although *E. asburiae* has been reported to possess autoinducer-2 (AI-2) receptors [15] there are no reports on the production of AHLs by this bacterial species. In this study, we aimed to identify the AHL(s) produced by the isolated Gram-negative bacteria *E. asburiae* from the *Enterobacteriaceae* family, obtained from Batavia lettuce.

## 2. Experimental Section

### 2.1. Sample Collection and Processing

Batavia lettuce samples were collected from a local market in Malaysia in a sterile container. The samples were processed within half an hour of sample collection. Briefly, 5 g of the leaf were placed in 50 mL of Brain Heart Infusion (BHI) broth and incubated overnight at 37 °C agitated at 200 rpm.

### 2.2. Isolation and Identification of Bacterial Strains

A 10 μL tenfold serial dilution (10^−1^, 10^−2^, 10^−3^, 10^−4^ and 10^−5^) of the overnight culture were plated on MacConkey agar. Bacterial isolates of interest were identified using a MALDI-TOF-MS (Bruker, Germany) [16] extraction method with UV laser wavelength of 337 nm and acceleration voltage of 20 kV. Each spot on the target plate was measured by the MBT-autoX.axe autoExecute method. The bacterial spectra were then analyzed in the Bruker MALDI Biotyper Real Time Classification (RTC) Version 3.1 (Build 65) software. The dendrogram was generated using the standard MALDI Biotyper MSP creation method.

### 2.3. AHL Detection of Bacteria Isolates

*Chromobacterium violaceum* CV026 which detects the presence of exogenous short chain AHLs ranging from four to eight carbons was used as an AHL biosensor. The bacterial isolates were screened using cross-streaking with CV026 [17]. *Erwinia carotovora* GS101 and *E. carotovora* PNP22 were used as positive control and negative controls, respectively [17].

### 2.4. AHL Extraction and Measurement of Bioluminescence

Bacterial colony that showed positive result for AHL detection via cross streaking with *C. violaceum* CV026 was incubated overnight in buffered Luria Bertani (LB) medium, pH 6.5, with 3-(*N*-morpholino)propanesulfonic acid (MOPS, 50 mM, pH 6.5) at 37 °C with shaking (220 rpm). The spent supernatant was then extracted thrice with an equal volume of acidified (0.1% v/v acetate acid) ethyl acetate. The extract was dried and stored at −20 °C prior to liquid chromatography-mass spectrometry (LC/MS) analysis.

Cell density bioluminescence measurements were done using an Infinite M200 luminometer-spectrophotometer (Tecan, Männedorf, Switzerland). Aliquots of 200 μL of diluted (1:100) *E. coli* strain [pSB401] overnight culture in LB supplemented with tetracycline (20 μg/mL) was added with 1 μL of extracted AHL to every well of a 96-well optical bottom microtitre plate [18,19]. The *E. coli* strain used harbors *lux* from the pSB401 plasmid [20]. Acetonitrile and synthetic 3-oxo-C6-HSL (250 pg/μL) were used as the negative and positive standards, respectively. Results were indicated as Relative Light Units (RLU)/OD495 nm against incubation time.

### 2.5. AHL Identification by Triple Quadrupole LC/MS

Extracted AHL was reconstituted in acetonitrile followed by LC/MS analysis using an Agilent 1290 Infinity LC system (Agilent Technologies, Santa Clara, CA, USA) equipped with an Agilent ZORBAX Rapid Resolution High Definition SB-C18 Threaded Column (2.1 mm × 50 mm, 1.8 μm particle size). The flow rate was set at 0.3 mL/min and the temperature at 37 °C. Injection volume was 2 μL. Mobile phases A and B used included water and acetonitrile (both containing 0.1% v/v formic acid). The gradient profile was set at A:B 80:20 at 0 min, 50:50 at 7 min, 20:80 at 12 min, and 80:20 at 14 min. Subsequent MS detection of separated compounds was performed on the Agilent 6490 Triple Quadrupole LC/MS system. Precursor ion-scanning analysis were performed in positive ion mode with Q3 set to monitor for *m*/*z* 102 and Q1 set to scan a mass range of *m*/*z* 80 to *m*/*z* 400. Molecular mass of *m*/*z* 102 refers to the lactone ring thus indicating presence of AHLs. The MS parameters were: probe capillary voltage set at 3 kV, sheath gas at 11 mL/h, nebulizer pressure of 20 p.s.i. and desolvation temperature of 200 °C. The Agilent MassHunter software was used for the MS data analysis to confirm the presence of AHLs. Analysis was based on the retention index and the comparison of the EI mass spectra with AHL standards.

## 3. Results and Discussion

### 3.1. Identification of Bacterial Isolates from Batavia Lettuce

**Figure 1.**
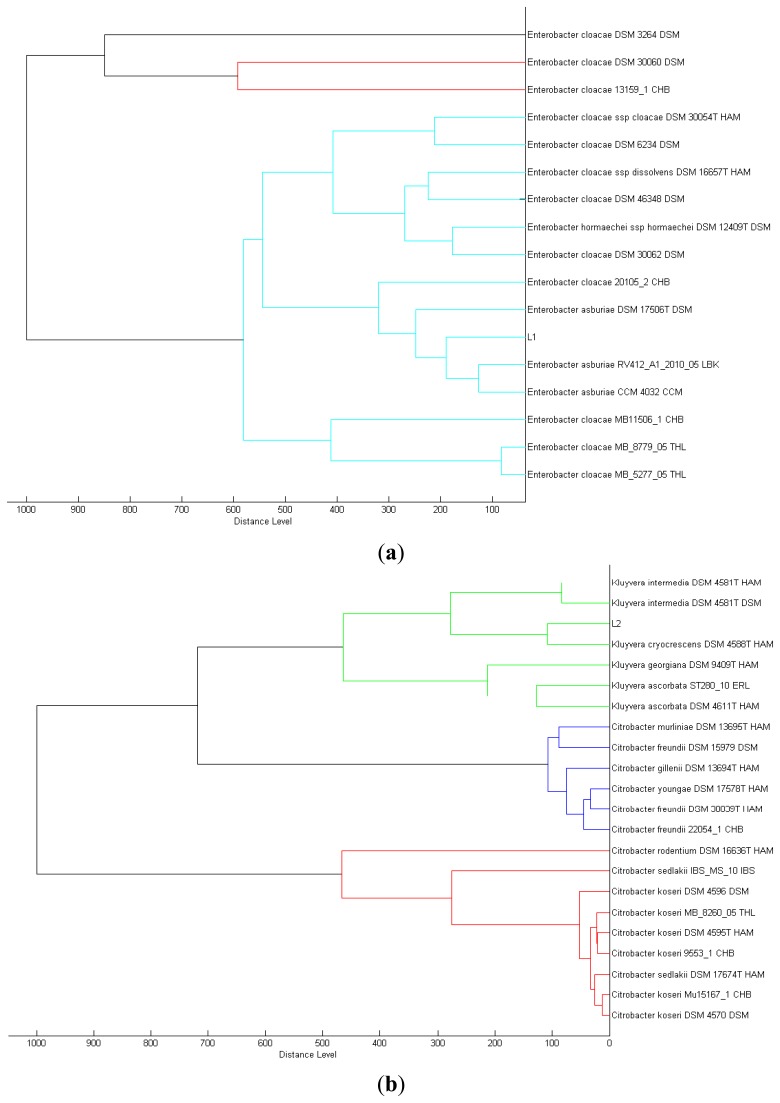

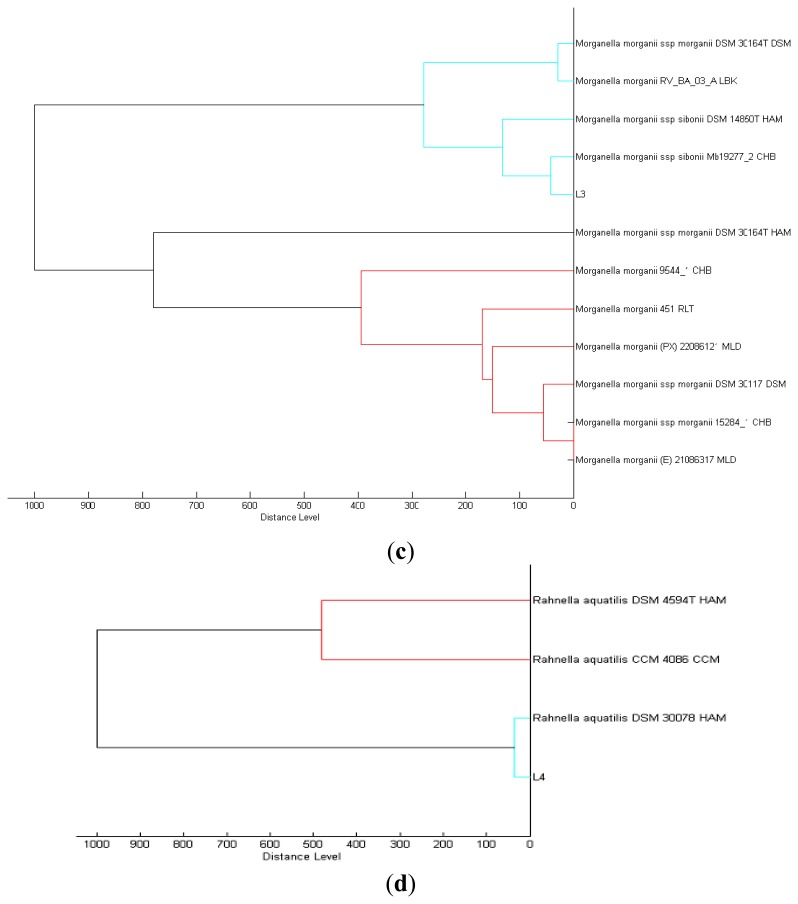
Phylogenetic positions of (**a**) L1, (**b**) L2, (**c**) L3 and (**d**) L4 isolates, visualized using the standard MALDI Biotyper MSP. The different colors of the branches represent distinct clusters among the organisms in the database.

We isolated a total of four different *Enterobacteriaceae* bacterial colonies from the Batavia lettuce sample. In order to identify the isolates, MALDI-TOF-MS was performed [21]. Two of the isolates could be identified to the species level with score values above 2.3, namely *E. asburiae* (L1) and *Morganella morganii* (L3). However, we were only able to identify the other two isolates, *Kluyvera* (L2) and *Rahnella* (L4) at the genus level with score values of 2.1 and 2.0, respectively [22]. Phylogenetic trees generated on all of the isolates are shown in Figure 1.

### 3.2. Production of AHLs by E. Asburiae

**Figure 2.**
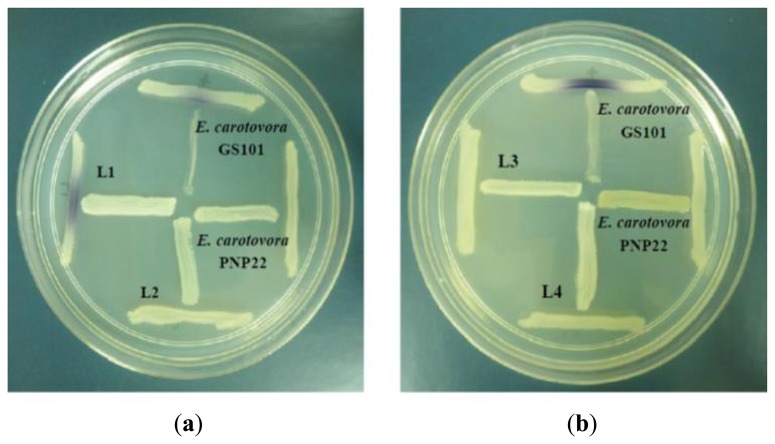
Screening for violacein production using *C. violaceum* CV026 cross streaking. *E. carotovora* GS101 and *E. carotovora* PNP22 were used as positive and negative controls, respectively. (**a**) Observation of purple pigment formation on the biosensor streak line indicates the production of exogenous short chained AHL molecules by the *E. asburiae* (L1) isolate; negative purple pigmentation for L2 isolate and (**b**) both L3 and L4 isolates indicate no AHL production from these isolates.

**Figure 3.**
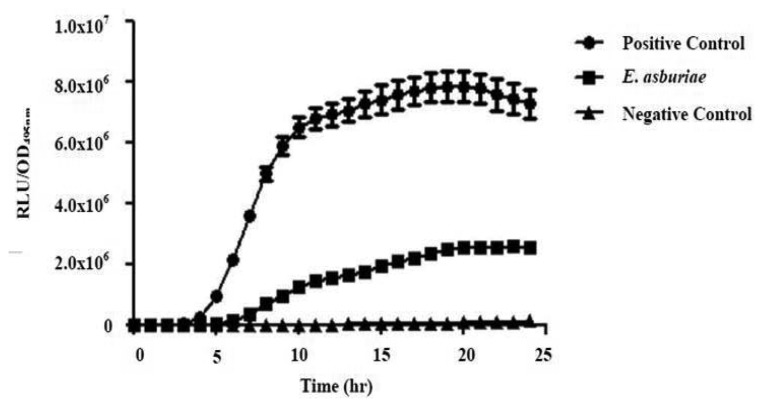
Detection of AHL production by *E. asburiae*. Bioluminescence measurement was done for 24 h, 37 °C growth in the presence of AHL extracted from spent culture supernatant of *E. asburiae*, synthetic 3-oxo-C6-HSL and acetonitrile as positive control and negative controls, respectively. Biosensor *E. coli* [pSB401] served as the biosensor. Data are presented as means of ± SEM values of triplicate experiments.

**Figure 4.**
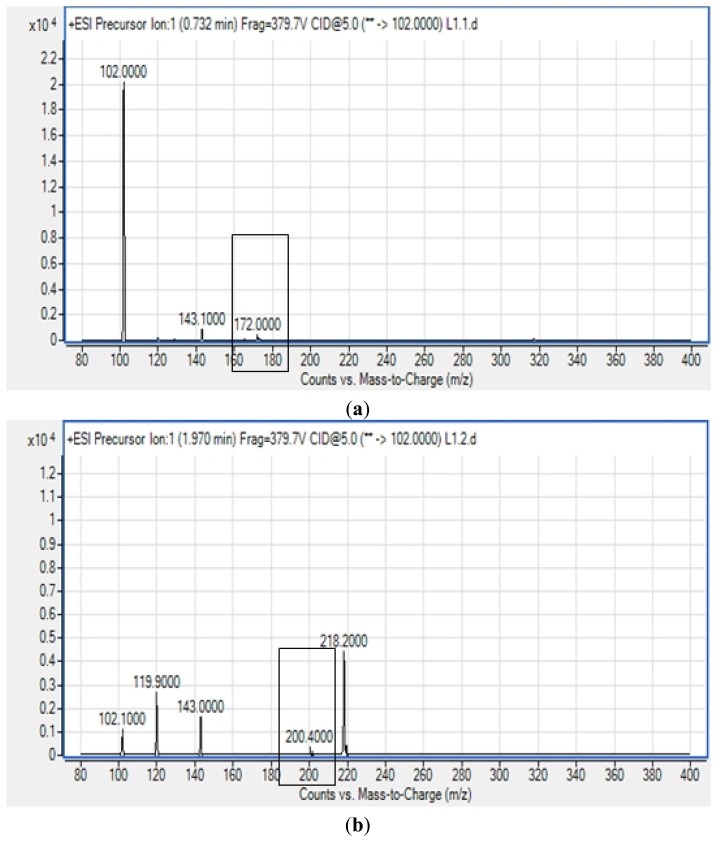
Mass spectra of the extracted AHLs from the spent supernatant of *E. asburiae*. (**a**) C4-HSL (*m*/*z* 172.0000) and (**b**) C6-HSL (*m*/*z* 200.4000) (boxed).

All four isolates of the Batavia lettuce sample were screened for the production of AHLs. Only *E. asburiae* (L1) was positive for the production of short chain AHLs (Figure 2), which triggered *C. violaceum* CV026 violacein production. The production of AHLs by the bacterium was further confirmed by using the luminometer-spectrophotometer, whereby the activation of bioluminescence of *E. coli* [pSB401] was observed (Figure 3). In order to identify the AHLs and to further confirm the production, high resolution triple quadrupole LC/MS system was used. The MS analysis results of the spent culture supernatant of *E. asburiae*, presented in Figure 4, provided evidence for the presence of C4-HSL (*m*/*z* 172.0000) and C6-HSL (*m*/*z* 200.4000).

*Enterobacteriaceae* colonization has been reported in numerous studies and is believed to cause the spoilage of food products [23,24]. Furthermore, members of the family *Enterobacteriaceae* have been indicated to cause gastrointestinal illnesses around the World and these outbreaks have been commonly connected to consumption of contaminated vegetable and fruit products [9,25]. This relation of both food spoilage and food-borne illnesses with enteric bacteria has caused an increase in multidisciplinary interest in researching the production of signaling molecules to better understand how these interactions may affect food safety. Hence, in this study, we focused on the isolation of the bacterial community from the *Enterobacteriaceae* family in fresh vegetable produce, specifically Batavia lettuce, which is commonly served as a garden salad.

We have isolated four different enteric bacteria from the lettuce sample. Members of the *Enterobacteriaceae* have been described as having AHL-mediated gene regulation [10,26]. This could have an important role for the food-borne *Enterobacteriaceae* community in terms of regulating the expressions of phenotypes and cellular responses such as secondary metabolite production, toxin production, competence, plasmid transfer and biofilm production [27–29]. Although *Morganella morganii* was isolated in this study and is a bacterium known to cause food spoilage and toxicity [30], it was found that this isolate did not produce any detectable AHLs, and among all the isolates tested in this work, only *E. asburiae* produced AHLs.

*E. asburiae* has been reported to cause competition to the growth of human pathogens *Salmonella enterica* and *Escherichia coli* O157:H7 [13]. Additionally, as reported previously the presence of *E. asburiae* could inhibit the growth of *S. enterica* serovar Newport and *E. coli* O157:H7 by more than 10- to 100-fold [9,31]. The system(s) involved in this antagonistic communication is still unclear. However, we speculate that the signaling molecules produced by *E. asburiae* in this study, namely C4-HSL and C6-HSL may play a part in inhibiting the proliferation of the surrounding enteric bacteria. Perhaps the AHLs regulate the production of exoenzymes or secondary metabolites that could act as an antagonists against other microorganisms. However, further study is required to verify the function of these AHLs produced by *E. asburiae* and their role in interspecies microbial growth suppression.

Recently, there is much interest in exploring QS as a novel anti-infectious therapy [32,33] because since it does not involve the use of antibiotics, theoretically this will reduce the drug resistance problem [32]. The discovery of QS in our *E. asburiae* isolate will allow the investigation of QS-mediated gene expression in this bacterium and also the development of novel anti-QS molecules [34–41].

## 4. Conclusions/Outlook

This study confirmed the production of AHLs in *E. asburiae* isolated from lettuce. The identified AHLs could be implicated in the regulation of physiological activities important in the survival of *E. asburiae* or other enteric microorganisms. Further studies are required to determine the importance and functions of these signaling molecules in the hope of enhancing produce safety and elucidating the mechanisms of QS-regulation in *E. asburiae*.
